# Hilda Mary Woods MBE, DSc, LRAM, FSS (1892–1971): reflections on a Fellow of the Royal Statistical Society

**DOI:** 10.1111/j.1467-985X.2011.01018.x

**Published:** 2012-07

**Authors:** Vern Farewell, Tony Johnson, Rosemary Gear

**Affiliations:** Medical Research Council Biostatistics UnitCambridge, UK; Medical Research Council Clinical Trials UnitLondon, UK; Palmerston NorthNew Zealand

**Keywords:** Hilda Mary Woods, History of medical statistics, Major Greenwood

## Abstract

We have previously described the content of a text by Woods and Russell, *An Introduction to Medical Statistics*, compared it with *Principles of Medical Statistics* by Hill and set both volumes against the background of vital statistics up until 1937. The two books mark a watershed in the history of medical statistics. Very little has been recorded about the life and career of the first author of the earlier textbook, who was a Fellow of the Royal Statistical Society for at least 25 years, an omission which we can now rectify with this paper. We describe her education, entry into medical statistics, relationship with Major Greenwood and her subsequent career and life in Ceylon, Kenya, Australia, England and South Africa.

## 1. Introduction

Hilda Mary Woods was the first author (with William Russell) of the first British textbook of medical statistics, published in 1931, 6 years before the renowned series of papers and subsequent book by Austin Bradford Hill. Almost nothing of her life was publicly known when the first two authors started research (Farewell and Johnson, 2010) on her book (Woods and Russell, 1931). They were fortunate, eventually, to find a close relative who provided details of the extraordinary life of this lady. They are honoured that Hilda's niece (and adopted daughter), Rosemary Gear, has joined them as a co-author on this paper which relies heavily on the personal memoirs and memories that she has provided.

Asterisks throughout the text reference an entry in a glossary which can be found in the on-line supplementary material for this paper.

## 2. The unheralded heroine

In the history of any scientific discipline there is a cast of characters who contribute in a variety of ways. Many will be largely forgotten whereas some will be the giants on whose shoulders subsequent progress is said to be based. Sometimes, though, the largely forgotten might usefully be remembered and Hilda Mary Woods, a Fellow of the Royal Statistical Society for at least 25 years, was such a contributor to the early history of medical statistics.

## 3. Early life

Hilda Mary Woods was born in 1892 in Doddershall*, Quainton, England, the daughter of William Ashburnham Woods and Mary Ann Woods (formerly Markham). She was the eldest daughter in a family of five surviving children: three boys and two girls.

Her early education was provided by a governess at home and included learning to play the piano. At 12 years of age she was sent to the Girls* High School in Northampton and boarded with a lady living close to the school as there were no boarding facilities at the school. Her abilities were evident in that she was moved up a year after her first term. After 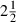 years she obtained her Junior* Oxford and Cambridge Certificate and several music certificates.

Meanwhile, her younger brothers had, in turn, been educated at home with a governess but were now ready to move on. Given the priorities of the age, they were sent to boarding school but that left no money for Hilda to continue in Northampton. She was therefore moved to Ellerker* College, Richmond, where one or two pupil teachers were taken in return for education and boarding. She was encouraged at the college to work hard academically as she did well in both music and general subjects. During this time she also went up to the Royal* Academy of Music to do the intermediate examination in piano and gained distinction. In December 1909 she gained her Senior* Cambridge Certificate with passes in arithmetic, religious knowledge, English, drawing and music, and went on to receive her Licentiate of the Royal Academy of Music (LRAM*) in piano with violin as her second instrument. With this background she hoped to pursue a music career.

Through her eldest brother, who was a student at Fitzwilliam Hall in Cambridge, she met his college friend, Frank Stevenson Long*. They were soon engaged. However, war broke out and Frank Long volunteered and was killed near Loos in France on September 26th, 1915. He was 25 and Hilda Woods was 23 years old.

## 4. The birth of a medical statistician

In her late 30s Hilda Woods wrote a short document concerning her start in medical statistics in 1916, titled ‘The birth of a medical statistician’. It is only appropriate therefore to allow this part of her life to be recounted in her own words.

‘Statistics: How dull says the man in the street. I can't think of anything more boring than columns and figures. Anyone can prove anything by figures. Only dry old professors and such ever become statis. no, I can't pronounce it, or spell it either! Even the man in the street is sometimes wrong. I am nearly forty, supposed to be quite good looking and my dressmaker seems to enjoy making me clothes because “madam carries them well”. I began work on statistics when I was a flapper*.

‘In June 1916 I wandered along Whitehall in search of work. My fiancé had been wounded and missing and now was reported killed. The world seemed very black and the future blotted out. Work was plentiful enough during those years of war and I was willing to accept anything. The Admiralty wanted any number of girl clerks starting at 25/- [shillings*] a week. The Home* Office, India* Office (one had to pass an entrance examination to be allowed to “clerk” in this austere building), all offered much the same pay. Finally I came to a door in Whitehall Gardens marked Ministry of Munitions and underneath in smaller letters the mysterious word Establishments. A porter directed me along endless passages, there was the tap-tap of typewriters everywhere, girls armed with files were hurrying to and fro. It seemed the hub of war activities in Whitehall.

‘I filled in a form and was interviewed. “Yes, girl clerks were wanted.”

“‘Oh, I see you have the senior Cambridge certificate. Are you any good at mathematics? There is a doctor in the Welfare Section who wants an educated person to travel round and get statistics from factories. You look too young tho’. What age are you?” I promptly added three years to my age and hoped silently I was educated but doubted it. However an appointment was made with the doctor for the following Monday. The pay offered was 27/6 a week and in addition, a subsistence allowance while travelling.

‘Full of the arrogance of youth my very indifferent knowledge of mathematics did not deter me. On Monday morning I went armed with my card of introduction for my interview, little thinking that the next sixteen years of my life would be spent working with this same doctor who became a world famous medical statistician. The day was one of London's dullest and greyest. The doctor's office was a draughty front room on the ground floor in a dismal narrow lane off Northumberland Avenue. The room was so dark that the electric light was perpetually on. Welfare work was of secondary importance in the terrific drive to produce ammunition for our men at the Front.

‘The doctor was a man in the early thirties wearing the uniform of a Captain* RAMC [Royal Army Medical Corps]. He had sharp penetrating eyes but received me with extreme kindness. I was shown some of the preliminary equipment of a statistician—Barlow's* Tables (a book full of columns of square roots and cube roots of numbers), a large brown book of Logarithms and a mysterious looking object called a calculating machine and Yule's* Elements of Statistics. My heart sank. My training in life had been principally in music. I held a diploma from the Royal Academy of Music and my ambition had been to become an accompanist to a singer, failing that, a teacher. Logarithms and music seemed poles asunder.

‘The doctor explained that after some elementary instruction in statistics he wanted an assistant to collect statistics from factories employing women in various parts of England. These visits might necessitate staying in one place for a week or two at a time.

‘“I am afraid” continued my prospective chief with a fatherly air, “you seem to me to be too young to travel about England alone. You would have to find your own lodgings and generally fend for yourself. I think you must discuss these questions with your parents before I can offer you the job. If they raise no objection you can begin work next week.”

‘The idea of travelling appealed to me greatly. It was no use consulting my parents because the answer would most certainly have been in the negative. Fortunately for me I never asked them although looking back I realise I was most ignorant of the ways of the world—my niece of fourteen is more sophisticated than I was in those days.

‘Monday morning saw me installed in the draughty office trying to fathom the intricacies of a calculating machine. It was an old model of a Brunsviga* known by the nickname of “Grandfather”—the beginner's machine.

‘“Grandfather” consisted of a drum-like cylinder with rows of knobs which had to be set to figures, a handle like that of a sewing machine to turn and a lower register to slide along for the results. There seemed so many movements to remember and every little while “Grandfather” would jamb. I assured my instructress I could work far quicker by hand. But like everything else which improves with practice, in a few days I could add, subtract, multiply and even divide on this marvellous machine. Figures soon became impressive. There is something rather thrilling in seeing the answer to say 879673.4×638942.7 turn up with a few turns of the cylinder and in less than five seconds. Such an instrument would gladden the heart of any schoolboy.

‘Many years later I had the honour of demonstrating on a similar machine to one of our Royal Dukes. Well I remember the polite but rather bored look on the face of the eminent visitors when introduced to the statistician's domain. But on being shown the machine the Duke's interest was immediately roused—“How does it work? May I try? I wish I had one of these.” while the eminent personages stood by and waited. The programme that day was delayed by about twenty minutes.

‘But I am digressing.

‘Mastering “Grandfather” seemed to me really an achievement. He was a noisy old fellow but the two other occupants of the room—the doctor and his assistant—were used to him and they too added to the noise tho’ in a lesser degree with their newer models. It has always surprised me how quickly the senses get accustomed to unpleasant sounds and smells when the mind is sufficiently concentrated on something of interest.

‘My next task was learning how to construct charts* and graphs. This was not my strong suit. The lines I drew in Indian* Ink always went “ragged” and varied in thickness. I have the greatest respect for good draughtsmen. The charts became exciting when I was allowed to enter up the weekly returns of sickness*. Some factories always seem to have a number of workers on the sick list while in others hardly anyone ever got ill. This led to much speculation and often enquiry. Were the conditions of work unhealthy in the former or were the records more correctly kept? Sometimes a factory would show a rapid increase in the sickness curve. Was this due to an epidemic? Perhaps flu?

‘The days passed quickly and pleasantly in spite of the dismal quarters we called our office. I sampled most of the nearest lunch places, Lyons*, ABC and others but found they were too dear for my slender purse so I brought sandwiches. I had to pay 15/- for lodgings and partial board out of my earnings (27/6) and there was 6d for daily bus fares. It was fun eating sandwiches along the Embankment and watching the gulls, until on one or two occasions an objectionable creature persisted in attentions and I fled to the security of our little room. I have spent many a lunch hour in the National Gallery. Tea was a pleasant break in the office brought in by a one-armed porter so like a wicked count he was known as Monty*. In the adjoining room, through which we had to pass inconveniently to the only lavatory, sat a real live general—a dear old gentleman who delighted us one day by appearing in full dress uniform complete with rows of medals. He was most apologetic and nearly walked into our store cupboard in his embarrassment.

‘After a couple of months in the office I was sent out on my first expedition to a large factory in a Midland town. The statistics I had to collect related to an enquiry into “wastage*” of labour. A large majority of girls working on munitions were unaccustomed to factory life and less stable than men. After a few months they got tired of one place and moved on to another factory thereby giving little return in output for the training received. The statistical investigation was an attempt to show to what extent this migration was true.

‘I felt very important when I set out with my bag complete with slips of cards on which to enter each worker's record. The factory authorities had been notified and on my arrival the manager handed me over to the staff manager and the welfare supervisor. The necessary arrangements were made for me to begin work the next morning.

‘In the meantime I had to look round for somewhere to stay. The hotels were either full or too expensive (my subsistence allowance was 7/6 a day). There was no room in the women's hostel. I began to feel anxious and tired trudging round with my bag which seemed to get heavier and heavier.

‘As a last resort I asked the way to the nearest vicarage. My luck was out. The vicar was away on holiday and a locum—a young bachelor—was in charge. I explained my difficulty. He said he was all alone in the large vicarage. There was plenty of room for me but he supposed he could not take me in. However he kindly took me to a neighbouring vicar and to my relief carried my case!

‘And now I come to one of those curious coincidences in life which incline one to believe in [Sentence incomplete in original]

‘I was taken to the house of a friend of the vicar's—a lady who had offered to take in guests. She was charming and gave me a most comfortable room. At dinner that night we were discussing the war and relatives who were serving. She mentioned that her husband, a major, was serving in a Worcestershire* artillery regiment. So was my eldest brother. We compared notes. Finally she produced a photograph. “Surely your brother can't be the Woods I have heard so much of from my husband?” He was! There in the group was my brother sitting beside her husband.

‘Although I have the happiest recollections of my first expedition it was a failure from the point of view of work. As so often happens in statistical work the records were not sufficiently complete for our particular enquiry. I had to act on my own judgement. I returned to London in a few days with a sample of my card slips filled with such data as I found i.e. as a proof of the inadequacy of these records. I was not expected back for at least two weeks and I remember the look of consternation on the face of the doctor when I walked into the office, inwardly trembling that as a beginner my judgement had been unsound. Fortunately for me the doctor approved of my action and forthwith made arrangements for my next journey.’

## 5. In the company of Greenwood

The doctor wearing the uniform of a captain in the Royal Army Medical Corps who hired Hilda Woods was Major Greenwood whose role in the development of medical statistics and epidemiology during the first half of the 20th century has been well documented. From 1916 until 1933, Hilda Woods worked alongside Greenwood.

Greenwood had been seconded to the Ministry* of Munitions during the war from the Lister* Institute where he was resident statistician. When he returned to the Lister Institute after the war he arranged to have Hilda Woods employed until the end of 1919 by the Institute at a salary of £150 per annum, ‘in order to continue the investigation of the relation between tuberculosis* incidence and industrial conditions’. Following on from a report on factories* by Greenwood, he and Hilda Woods co-authored a report of the Industrial Fatigue Research Board in 1919 titled ‘A report on the incidence of industrial accidents upon individuals with special reference to multiple accidents’ (Greenwood and Woods, 1919). This report was frequently referenced in Royal Statistical Society discussions of papers on accidents in subsequent years, the latest occurrence found being in 1949. On September 1st, 1919, Greenwood moved to the newly created Ministry of Health and Hilda also resigned from the Institute to move with Greenwood to become part of the Ministry of Health, Statistical Services. When Greenwood was seconded from this role to the Medical Research Council's (MRC's) National* Institute for Medical Research in Hampstead, North London, Hilda Woods was seconded along with him.

While there, she was involved in a variety of studies and was a co-author on four publications during her time there. However, she was also Administrative Assistant under Greenwood and Sir George Buchanan* at the National Institute for Medical Research and various letters to her from Greenwood during this period indicate that she was a central cog in the administration of Greenwood's activities. For example, in a letter of September 3rd, 1923, Greenwood writes to ‘Dear Miss Woods’ and asks her to deal with a variety of matters, including the simple posting of a letter and paper, phoning the Research Department of the Labour Party saying that she is speaking personally for Greenwood and not the Ministry, and asking her to consider various options for dealing with a short staffing situation. It is noteworthy that Greenwood also writes that

‘I don't think *we* [our emphasis] can carry on for more than another year on the present basis, there is really too much for us to do’

and

‘If you on the official work and Miss Newbold* on the mathematical work fell ill, chaos would descend upon us at once’.

It is clear that Greenwood saw Hilda Woods as very much part of a collaborative team.

In 1924 Hilda Woods did fall ill and Greenwood writes to ‘Dear Miss Hilda’‘to get as fit as possible, however long it takes’. In a letter 3 days later Greenwood writes of a theatre outing with his wife, Rosa, saying ‘Had you been with us, yesterday evening would have been a perfect joy’ while also reporting on the computing situation:

‘The second oldest Brunsviga has gone on strike and Brunsviga people are rather pessimistic about its recovery …. I expect that it is true it has had more wear than any of the others, grandpa B. being smaller was not so much employed. Grandpa by the way seems fairly well. I hope we shall manage to get a new machine out of the MRC*.’

He also writes concerning a possible move of their team that

‘Perhaps, in any event, we shall do better where we are for in the fullness of time there will be an amalgamation of the MRC and Ministry of Health statistical services, if we just wait quietly’.

It is clear from this correspondence that Hilda Woods was both personally close and professionally invaluable to Major Greenwood. It is not surprising therefore that in a letter to ‘Dear Miss Woods’ on September 2nd, 1926 (a year or so before Greenwood moved to the London School of Hygiene and Tropical Medicine (LSHTM)), he writes

‘It is important in your interests as well as mine that you should be the only commissioned member of the Ministry Staff to accompany me to the School, …’.

A few months later, Greenwood indicates that he will take Hilda Woods's advice to take a break and finishes the letter with

‘Good bye now and many thanks, you and Newbold are the only people who keep me from throwing my hand in’.

Another indication of Hilda Woods's professional development is perhaps indicated by her election in 1926 to Fellowship of the Royal Statistical Society. Her election was proposed by Major Greenwood and seconded by T. H. C. Stevenson, the Superintendent of Statistics at the General Register Office from 1909 to 1933.

## 6. To the London School of Hygiene and Tropical Medicine

On February 21st, 1928, Greenwood and Woods transferred to the Division of Epidemiology and Vital Statistics at the LSHTM. They were the only whole time officers of the Division. Hilda Woods was appointed as Assistant Lecturer on the permanent staff of the University of London and the first female lecturer at the LSHTM. As well as giving a regular course of lectures on epidemiology and vital statistics to medical postgraduates taking the Diploma in Public Health course, she took on the duties of Administrator of the Division and the responsibility for organizing, and being in charge of, practical classes.

While at the LSHTM, Hilda Woods published papers on a variety of diseases, including respiratory disease (Woods, 1927, 1928a), scarlet fever and diphtheria (Woods, 1928b), as well as one methodological paper (Woods, 1929) that compared analytic and graphical methods for interpolation in life tables. In Greenwood's Division report of 1933, the last year that Hilda Woods was at the LSHTM, he writes

‘The most important published work from the Department during the year is certainly Dr. Hilda M Woods’“Epidemiological Study of Scarlet Fever in England and Wales since 1900”, …’

(Woods, 1933). This quote also reflects the fact that Hilda Woods was awarded her doctorate of science (London) in vital statistics on May 17th, 1933, in the Faculty of Science as an internal student. This was her sole university degree and she had come a long way from the musical flapper of 1916!

As the first female lecturer at the LSHTM, Woods was blazing new ground and, to give some idea of this, the recollections of her niece (and adopted daughter) serve well.

‘Mum used to tell stories of the trouble she had with each new intake of post-graduate students. They were all male and some obviously older than her, or at least they looked it, and at the first lecture of each intake they would universally slam down their pens, cross their arms and put their feet up on the desk when she walked in to give her lecture. What could such a young person and, worse, a female know that they did not? By the end of their course she would have a queue outside her office asking her to please help them with all sorts of statistics problems. She used to ask them why they thought she should help since they had been so rude at her first lecture. ‘Here is another story about the School. When the Division moved to Keppel* Street she went on the first day to the Staff Common Room for morning tea. She was confronted at the door by a male lecturer who informed her that HER common room was down the hall and this was not where she should be. “Down the Hall” was where the female secretaries and clerks were. Needless to say he was mortified when he was informed that he had made a mistake.’

Another project taken on by Woods at the LSHTM was the writing of a book, *An Introduction to Medical Statistics*, co-authored with her colleague William Russell and first published in 1931 (Woods and Russell, 1931). This book, which was primarily directed towards the Diploma in Public Health students in the LSHTM, has been examined in some detail elsewhere (Farewell and Johnson, 2010), particularly in comparison with Hill's well-known text *Principles of Medical Statistics* (Hill, 1937). The final paragraph concludes

‘In contrast to Hill's *Principles of Medical Statistics*, Woods and Russell's *An Introduction to Medical Statistics* is comparatively unknown. However, it is no disservice to Hill to bring this lesser known volume out of obscurity since it deserves to be recognized as reflecting an important stage in the transition from vital statistics to medical statistics. Indeed, while it is limited in its content, it can be regarded as in the forefront of texts on medical statistics as the subject is now understood. Given the background of the two authors, this is a particularly considerable achievement. Their ability to move from relatively routine vital statistics to broader interests was fostered in the environment of the LSHTM and by Greenwood's leadership of the work there. But as in all such situations, the individuals involved had to take advantage of the opportunity and their work is a testament to their willingness to take on what must have been a particular challenge.’

As well as her scientific contributions, the level of administrative responsibility that Woods had is reflected in a note from Greenwood on November 31st, 1931. He writes

‘I shall be glad if, during my absence, you will deal with the administrative and official matters that I normally deal with as head of the division’.

Hilda Woods's tenure at the LSHTM came to an end in 1933 following her engagement to Roger Fowke*. In a letter to ‘My dear Doctor’ on May 27th, 1933, Greenwood reflects on her departure.

‘You take away with you another great piece of my life; when you came to me I was a young man and of the successes and sorrows of my life you have been a partner; we have moved from junior “temporary” officer and “flapper” to university professor and doctor and done so honourably and without favour’.

And some flavour of their joint efforts is perhaps reflected in the additional comment

‘I shall at least do my best to look after our little flock although the womanly intuition has been taken from us’.

It can be noted that the salutation on subsequent letters from Greenwood to Hilda were predominantly ‘My Dear Portia*’. After Greenwood, his wife and Hilda attended a production of *The Merchant of Venice*, Portia became his nickname for her.

**Fig. 1:**
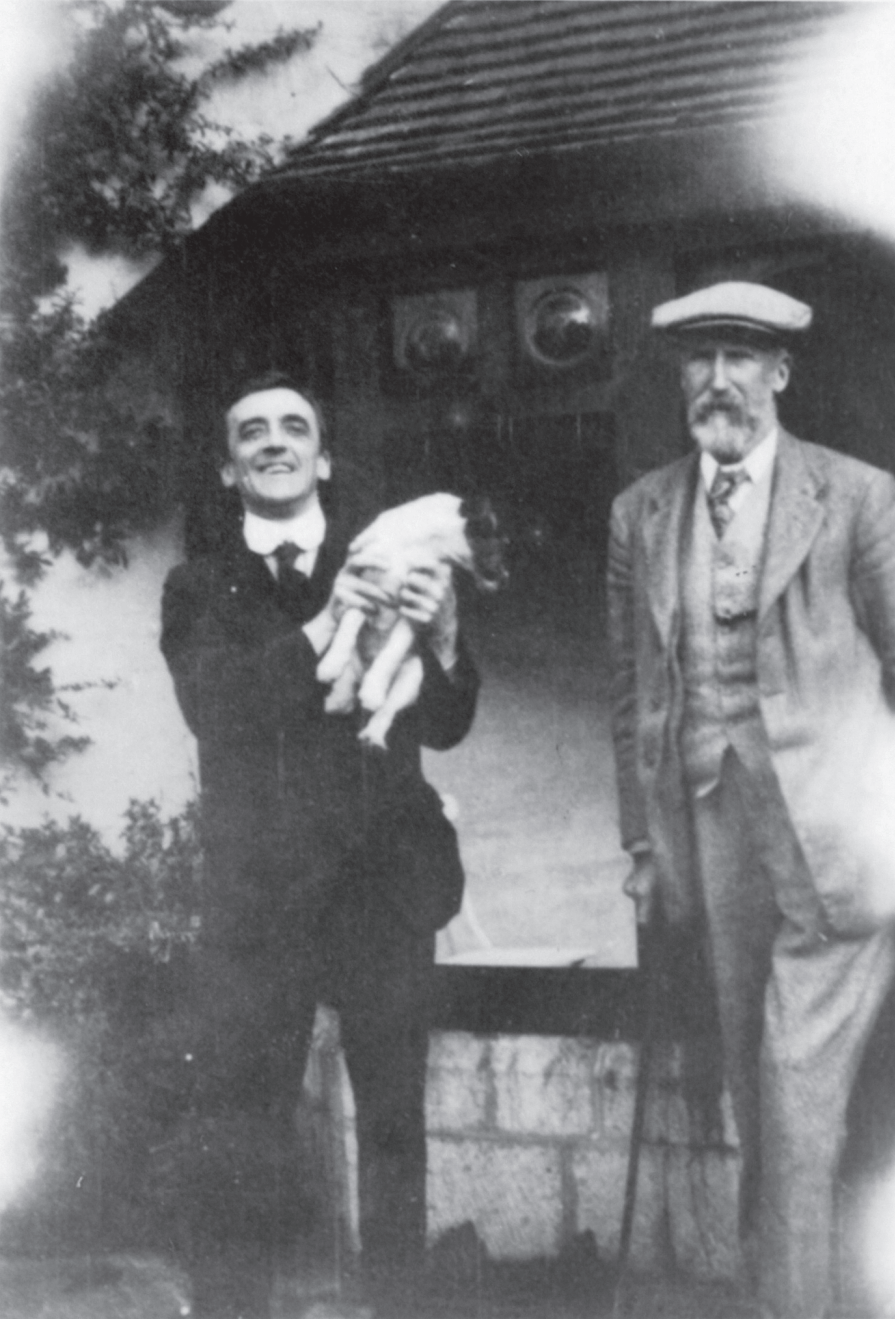
Major Greenwood and William Ashburnham Woods (reproduced by kind permission of Rosemary Gear)

**Fig. 2:**
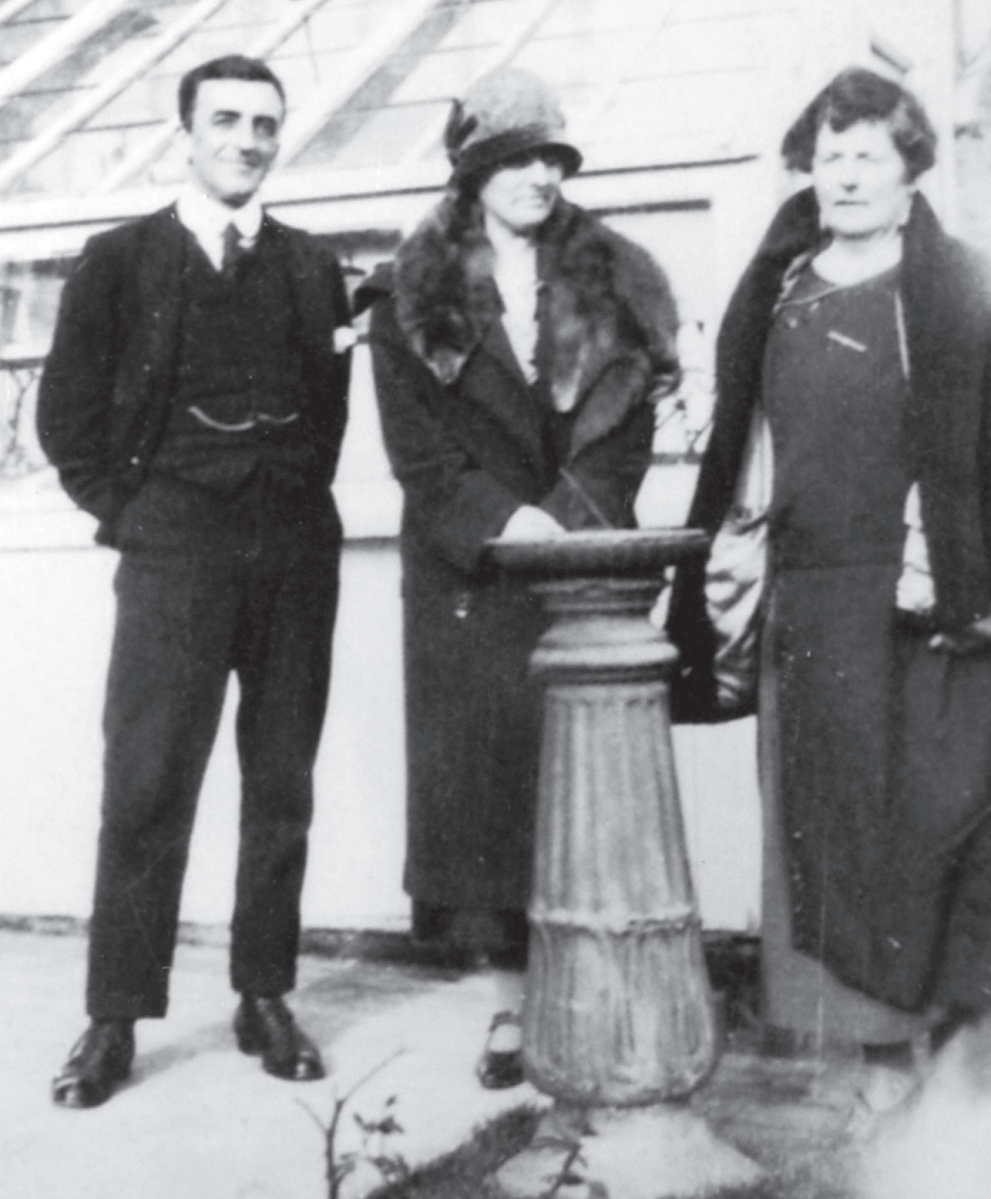
Major Greenwood, Rosa Greenwood and Mary Ann Woods (reproduced by kind permission of Rosemary Gear)

Despite their close relationship over the 30 years from 1916 to 1946 they published just two joint papers: one the extensive report on the incidence of industrial accidents in 1919, and one on the thymus in 1927. Maybe this is a tribute to Greenwood's tutelage of his staff and the resulting confidence that he had in them. However Hilda's ‘integration’ into Greenwood's family is apparent in their correspondence and in 1925 she was third author of a paper on heights and weights of patients in mental hospitals published in *Biometrika*; Rosa Greenwood, Major's wife, was the first author, and the second was Thompson, and not Greenwood himself (Greenwood *et al.*, 1925). At the same time Greenwood's association with Hilda's family is evident in a photograph of him with her father (Fig. 1), and in another of him with her mother and Rosa (Fig. 2).

Greenwood also much appreciated Hilda's musical talent for, as the third author reflects,

‘As Head of his department he quite often had get-togethers to mark special occasions such as milestones, significant anniversaries, and his FRS. At these Mum would always play the piano and other members of staff would sing and do musical skits. Greenwood also had a grand piano at his home and Mum often visited the family to play.’

The appreciation extended far beyond the department for

‘At the congratulatory dinner to Sir Andrew and Lady Balfour* in January 1930, Mum was part of the after-dinner programme which was all music’.

## 7. Ceylon

Roger Fowke was a prominent businessman in Ceylon and therefore Woods's break with Greenwood was not simply associated with marriage but with a marriage that would take place at St Andrews Church, Colombo, Ceylon, on Saturday, December 9th, 1933. However, having lost her *fiancé* 18 years earlier, Hilda Woods suffered yet another tragic loss when her newly wed husband, aged 53 years, died suddenly, from a tooth abscess which led to *septicaemia*, 2 months later in February 1934.

This second tragedy so similar to the first but this time in a very new land thousands of miles from home, without family for support even by telephone, and with very limited social contacts, must have been a great ordeal. Many surely would have abandoned the new life and returned home in despair. But not Hilda; the strength of character and resolve that drove her accomplishments in previous years must have been sorely tried by this event but she again emerged triumphant after immersing herself in important medical and social services of various kinds in Ceylon.

Hilda Fowke was a member of the Ceylon Government Commission appointed in November 1934 by the Minister for Labour, Industry and Commerce, Ceylon, to study factory conditions and to advise on a new Factory Act. Also in 1934 she was temporarily appointed to the Medical Department, Ceylon Government. In this role, she helped to compile and draft, as part of the Ceylon* Government Commission on Malaria, a report on the epidemic, including statistical records. Both these appointments led to reports tabled in the British and Ceylon Parliaments. The malaria epidemic in Ceylon was significant and Hilda Fowke also organized temporary hospitals during the height of this epidemic for the Medical Department of the Ceylon Government. During 1935–1937 her appointment to the Medical Department was further extended to organize a new department for studying the prevalence of various diseases in Ceylon.

It can be presumed that Greenwood maintained contact with Hilda during this period but only two letters have been preserved. The beginning of a letter of January 16th, 1936, reflects their relationship and is a further tribute to her character.

‘Your letter was very welcome. I thought the part of the blue* book on Malaria, which was your work, excellently done and I have not the least doubt that what you are doing will be of solid benefit to science and the public service. God (you will forgive me for using these old fashioned ways of speaking, for after all I am nearer sixty than fifty now) has tried you severely, but He has permitted you to use your talents and I firmly believe that in the using of them you will find peace and the happiness which comes, as happiness always comes, unawares.’

The number of her roles grew as she lectured on Social Economics at University College, Colombo, organized infant and child welfare clinics in Colombo, was Honorary Secretary of the Child Protection Society and the Ceylon Red Cross Society (at its inception) and was Honorary Treasurer of the Ceylon Girl Guides.

On June 23rd, 1936, she was awarded Membership of the Order of the British Empire (Colonial Office List) for social services in Ceylon.

## 8. War returns

In early 1936, Hilda had become very ill with supposed ‘heart problems and toxaemia’. Failing to recover on a recommended 2-month sea voyage to China, she was sent to England where Sir Thomas Dunhill* diagnosed her retro*-sternal goitre. Because he was loath to operate at that time, Hilda returned to Ceylon in March 1937. However, she ultimately had to return to England in February 1938 for an operation. Although she intended to return to Ceylon, war broke out before she was sufficiently well to travel and a new phase of her life began.

A letter exists from Greenwood, dated October 24th, 1936, when Hilda Fowke was in England, and refers to her ‘slow convalescence’. The reminiscences recorded earlier in this paper might well have been prompted by Greenwood who also wrote in this letter

‘Why not do some writing? You have had varied experience. Write your reminiscences. At worst it will interest you, and it might interest others.’

Hilda Fowke did recover and in June 1940 she was called on by Professor Jack Drummond*, Scientific Advisor to the Ministry of Food, to oversee some investigations concerning vitamins and minerals. She undertook a variety of experiments with fortified food, bread, chocolate, margarine and other foodstuffs with the co-operation of factory canteens, institutions and mothers; one big experiment related to rosehips. In addition to these studies, part of her role was to visit factories to speak to the workers about the importance of nutrition.

**Fig. 3:**
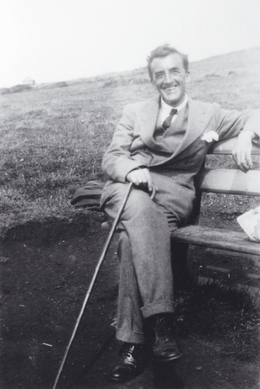
Major Greenwood, relaxed and smiling (reproduced by kind permission of Rosemary Gear)

**Fig. 4:**
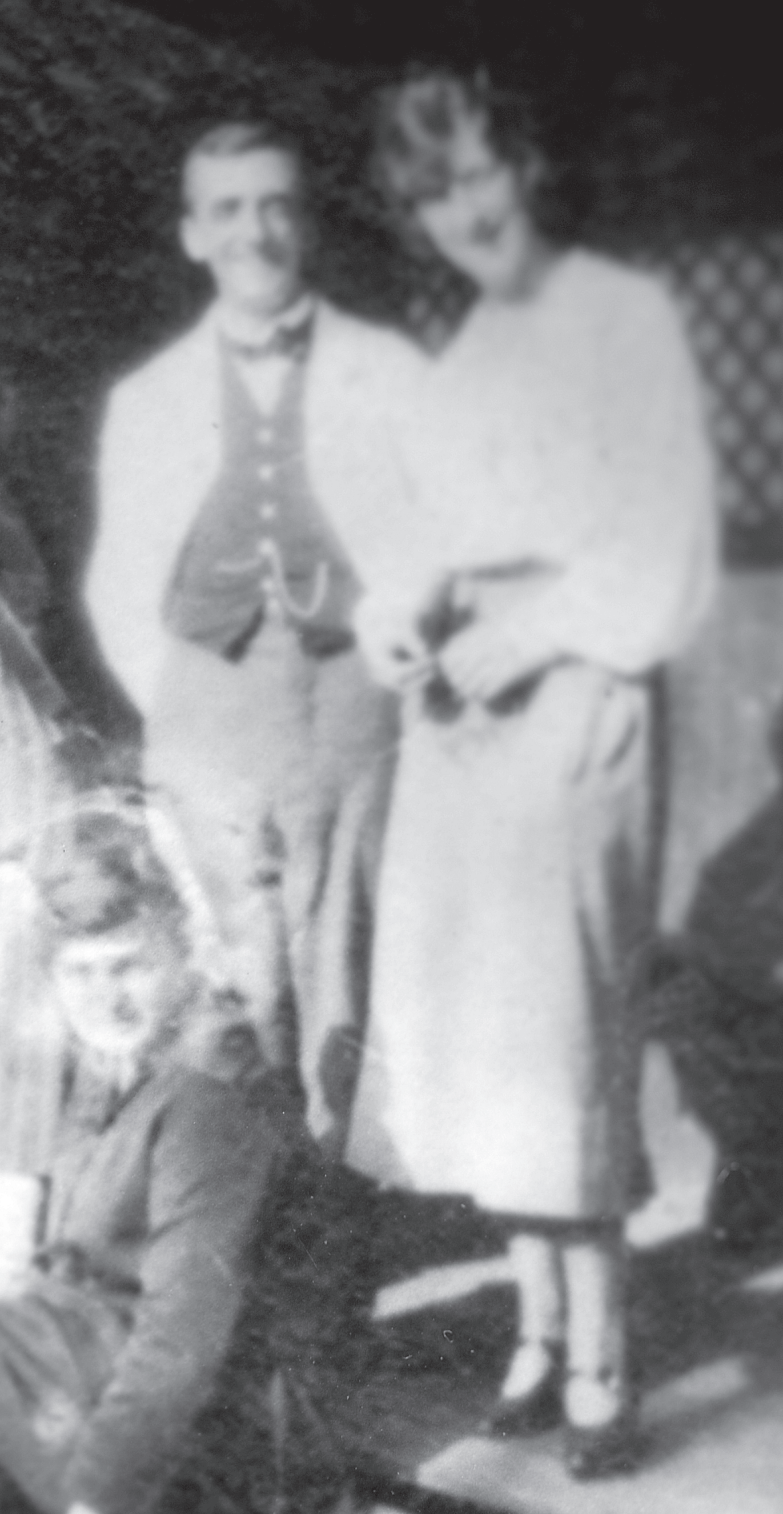
Major Greenwood with Hilda Woods (extracted from a family photograph by kind permission of Rosemary Gear)

Only one formal publication, in the *British Medical Journal* (Fowke, 1943), and single authored by Hilda Fowke, appears to have arisen out of this activity: remarkably a multicentre study in which girls at five orphanages were assigned ‘at random’ to either fortified* chocolate or ordinary chocolate; her personal papers indicate that, during this period, she did collaborate with Major Greenwood on statistical aspects of her work. She became a member of the Nutrition Society, no doubt prompted by her war work, and as a discussant of one of his papers presented at a conference on nutrition in 1945 reveals that she was involved in the first* dietary survey made by the Ministry of Food; Hill was also a discussant. Fig. 3 shows a relaxed and smiling Greenwood and Fig. 4 (extracted from a family photograph) shows Greenwood and Hilda Fowke; both pictures may have been taken around this time. Perhaps less related to her work but reflective of her other activities, she was also a member of the United Nations Relief and Rehabilitation Organization from 1943 to 1945, and joint President of the Whitchurch* and District Nursing Association in 1945.

## 9. Leaving medical statistics

When an outbreak of foot-and-mouth disease in Buckinghamshire forced the destruction of Hilda Fowke's youngest brother's herd of dairy cows, he emigrated to Kenya. However, his wife, Nancy, struggled with living in Africa and he appealed to his sister to come out to Kenya to help with the adjustment. Hilda was intermittently in Kenya from June 1946 to the end of May 1947 and it was May 31st, 1947, that, to some extent, defined the rest of her life. On that day, Nancy Woods gave birth to a daughter but died 7 hours later. The devastated father appealed to Hilda for help and, for the third author, Hilda

‘became my mother from when I was discharged from hospital at two weeks old’.

Her new commitment was complete. Thus, when she was offered a job by Dr Maclennan* at the Medical Department, Nairobi, in August 1947 on the recommendation of Sir Rupert Briercliffe* with whom she had worked in Ceylon during the malaria epidemic, she declined.

Although no longer working in medical statistics, Hilda remained a member of the Royal Statistical Society until at least 1951, according to the membership lists for that year. The subsequent membership list available, for 1961, did not include Hilda Fowke.

**Fig. 5:**
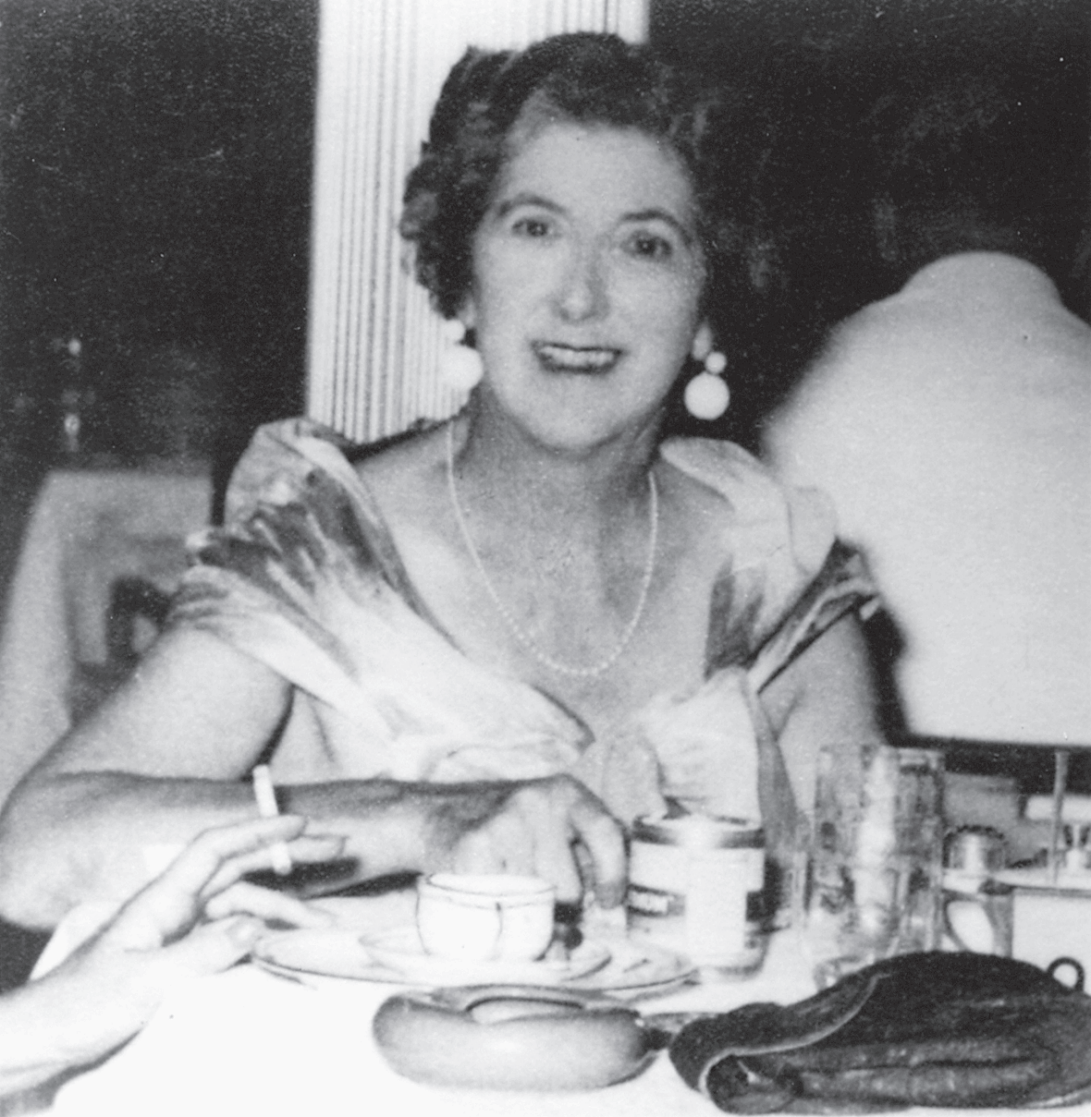
Hilda Fowke (Woods) in the 1950s (reproduced by kind permission of Rosemary Gear)

Over the subsequent years, attempts at farming were made in New Zealand and Australia and, finally, in Rhodesia. However, in 1958 Hilda's brother died and a few years later she returned with Rosemary to England, where they stayed until 1970. Throughout this time, Hilda Fowke was heavily involved in various volunteer roles and took a keen interest in current affairs, with a particular concern for social issues. Fig. 5 was taken in the 1950s.

When the third author, following in Hilda's footsteps, was offered a medical research job in South Africa in 1970, ‘Mum of course came with me’. And it was in the Transvaal that, after a series of strokes, Hilda Fowke (*née* Woods) died on November 29th, 1971, just a week before her adopted daughter was married to the son of a student (James Gear*) that Hilda Woods had taught at the LSHTM 40 years earlier.

## 10. A final reflection

To a large extent, the life of Hilda Woods speaks for itself. Out of great personal loss, and taking advantage of a wartime opportunity, she became a primarily self-taught medical statistician who, predominantly behind the scenes perhaps, made a major contribution to medical statistics in the UK in the first half of the 20th century. Her science may not have been the stuff of giants, but she blazed new trails, both for the subject and for the women in it. She deserves to be remembered for this but in perspective. Major Greenwood wrote to Hilda Woods before she left England in 1933

‘You have the consciousness of work well done and of more important work still to do. … Teaching, research, organising, these are indeed of some value, I hope and believe of considerable value, but they are, after all, only the secondary duties of human life. The world struggled on before there were any professors of statistics or schools of hygiene; when family life ceases to be the *summum bonum* of human experience then indeed the end of all things is near, ….’

The family life which occupied the later half of Hilda Woods's life was far different from that Greenwood, or she, might have imagined. Its value is perhaps best reflected in the memories of the third author, her adopted daughter.

‘Mum was an amazing person. She had a deep and private faith which helped her through many sadnesses and traumas. I never heard her say an unkind word about anyone and she was intensely loyal to her friends and family. She was the “matriarch” of the Woods family and kept in close touch with all her siblings and their offspring and her numerous cousins. She never gave advice unless asked and then was very measured in what she had to say. I did not realise until I went through her papers that her work had involved so much organising and administration but in all my memories of her she was always doing some volunteer work and it always involved organising!

‘She treated everyone equally and never hesitated to help if needed. I remember going with her to some shops in Salisbury (Rhodesia) when I was about ten. There was a huge commotion behind the buildings with a crowd of people milling around and very agitated. Someone had been stabbed. My mother never hesitated. She told me to stay put and with no back up she pushed her way through the throng and helped the victim until the ambulance arrived. No one else dared get involved and it was a very dangerous situation but Mum never worried about that.

‘She loved all animals and used to ride side-saddle until, in her early twenties, she had a very bad hunting accident which damaged her spine. She was unable to play tennis after that and also could not walk long distances. Whenever circumstances allowed she had dogs. When she lived at Hardwicke* after her operation in 1938, she had a black Labrador, Flash, who ran in front of the plough. The vet said she had to be put down as she was too badly injured but Mum refused and nursed her back to health. She had to change her dressings and check on her every four hours, day and night, until she recovered. Some while later she had pups which vindicated Mum's decision. She was a very knowledgeable and keen gardener. She created a lovely garden when we lived in Suffolk* and apparently had a beautiful walled garden at Hardwicke. We always had flowers in the house and when it was her turn for decorating the Church the flowers all came from her garden.

‘She was always very smart and when I knew her she used to have two outfits made in spring and again in autumn. As a teenager I loved going to buy the material and choosing the design!’

From any perspective, her's was a life well lived!

## Supporting information

Additional ‘supporting information’ may be found in the on-line version of this article:

‘Hilda Mary Woods MBE, DSc, LRAM, FSS (1892-1971):Reflections on a Fellow of the Royal StatisticalSociety’.
