# Supplementary material for: Hilda Mary Woods MBE, DSc, LRAM, FSS (1892–1971): reflections on a Fellow of the Royal Statistical Society
Source: J R Stat Soc Ser A Stat Soc. 2012 Jul;175(3):799–811. doi: 10.1111/j.1467-985X.2011.01018.x (PMC3437511; doi:10.1111/j.1467-985X.2011.01018.x)
Supplement: Supplementary file 1 [file rssa0175-0799-SD1.pdf]

Supplementary Information

Hilda Mary Woods MBE, DSc, LRAM, FSS (1892-1971): Reflections on a Fellow of the Royal Statistical Society

Vern Farewell, Tony Johnson, and Rosemary Gear

Glossary

[References to entries in this glossary are marked by asterisks in the main text. They have been compiled from many websites, especially Wikipedia, and other sources. We have constructed it as an aide to understanding institutions, places, and people mentioned in the text.]

Balfour, Sir Andrew, KCMG, CB, LL D (1873-1931)
A Scottish Medical Officer who specialised in tropical medicine spending twelve years in Khartoum, Sudan. He is noted as a medical author and also wrote historical adventures, mainly set in Scotland. In 1923 Balfour was appointed Director of the London School of Hygiene & Tropical Medicine, and supervised the construction of a new building for the school. In 1929 he suffered a nervous breakdown, believed to have been caused by the pressures of his new post. In 1930 he was admitted to hospital to be treated for clinical depression. Tragically his frozen body was found on 30 January 1931 in the grounds of the hospital after he had fallen from a window.

Barlow's Tables
(of squares, cubes, square roots, cube roots, reciprocals of all integer numbers up to 10,000). A collection of useful numerical tables compiled by Peter Barlow (1776-1862), first published in 1814, and frequently thereafter.

Blue Book
An official publication by the UK government that sets out an extended explanation of its policy; it is bound in a blue cover and contrasts with a white paper that is a government report on a specific subject and less detailed. However we think Greenwood's reference is to the report produced for the government of Ceylon (it had a blue cover). (See *Ceylon Government Commission on Malaria*, below).

Briercliffe, Sir Rupert CMG, OBE, BSC, MD, FRCP, DTM&H (1889-1975)
Graduated in medicine in 1910, took the DPH in 1912, and was appointed Medical Officer of Health Manchester in 1913. During the First World War he was in the Royal Army Medical Corps. In 1919 he became Principal Medical Officer at Haifa and from 1920 to 1930 Deputy Director of Health, Palestine. He then went to Ceylon, where he was principal of the Ceylon Medical College until 1936 when he became Director of Medical Services, Nigeria. From 1940-46 he was medical adviser to the Controller for Development and Welfare in the West Indies.

Brunsviga

A brand name of a popular series of calculating machines produced by the German manufacturer Grimme, Natalis and Company from the 1890s onwards. We cannot be certain about the models used by Hilda Woods and Greenwood. The picture below shows the model MD manufactured between 1911 and 1926; its dimensions were 35x15x12 cm and weight 8.7 kg. [Source: W Szrek].

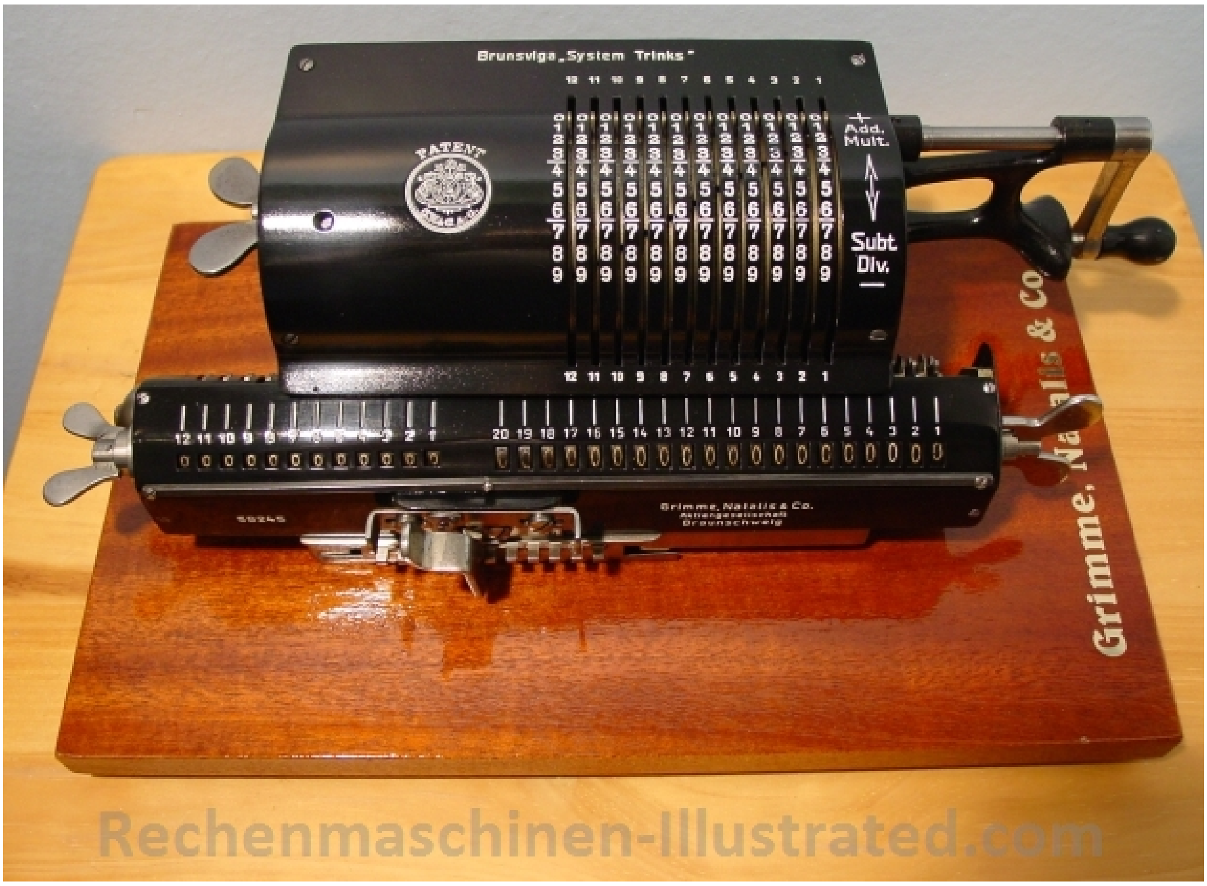

Buchanan, Sir George Seaton, CB (1869-1936)
Eldest son of Sir George Buchanan, FRS, who was principal medical officer to the Local Government Board from 1880 until 1892. The younger Buchanan graduated at the University of London and later became a fellow of the Royal College of Physicians. In 1895 he was appointed a medical inspector in his father's old Department. After serving for nearly forty years in this department and the Ministry of Health, which was created from it, he retired in 1934.

Captain RAMC
Major Greenwood served as a Captain in the 1st London Sanitary Corps, Royal Army Medical Corps, during the First World War.

Ceylon Government Commission on Malaria
In 1934-35 Ceylon suffered its worst epidemic of malaria and emergency measures had to be taken both to understand its transmission, and to set up medical services. A Commission was set up by the Government to report on the epidemic and the title page from the resulting publication is shown below. The Director of Medical and Sanitary Services at that time was R Briercliffe (see above) and he signed the report; Mrs R Fowke (Hilda Woods) D.Sc. is listed among the 37 contributors.

SESSIONAL PAPER XXII.—1935.

The Ceylon Malaria Epidemic, 1934-35.

Report by the Director of Medical and Sanitary Services.

SEPTEMBER, 1935.

Printed on the Orders of Government.

PRINTED AT THE CEYLON GOVERNMENT PRESS, COLOMBO.
To be purchased at the GOVERNMENT RECORD OFFICE, COLOMBO; *price Rs. 4·50.*
1935.

The comprehensiveness of the report is demonstrated by its contents also shown below. In addition there is a supplement of exquisite coloured maps and line charts which shows the geography of the island and the progress of the epidemic by region.

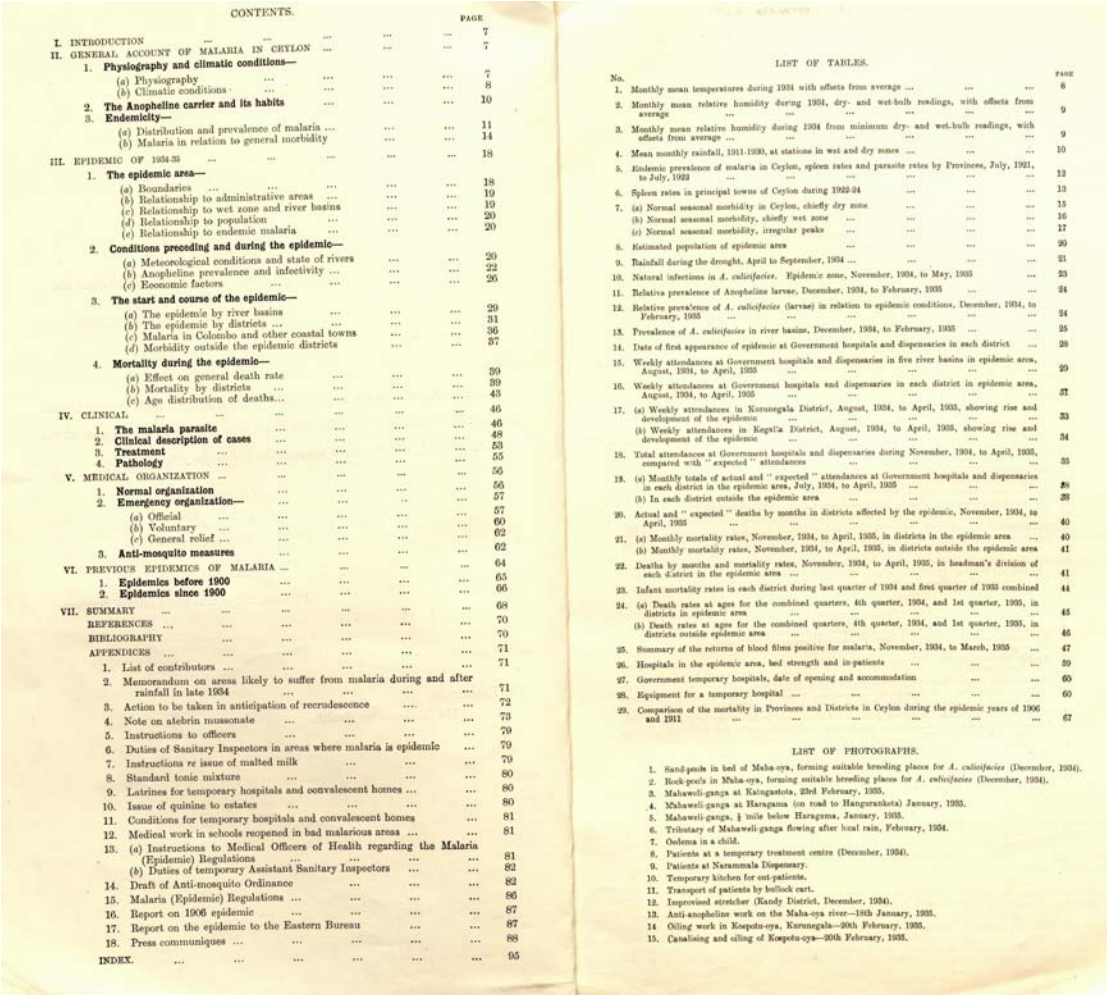

CONTENTS.

PAGE
I. INTRODUCTION ... 7
II. GENERAL ACCOUNT OF MALARIA IN CEYLON ... 7
1. **Physiography and climatic conditions—**
(a) Physiography ... 7
(b) Climatic conditions ... 8
2. **The Anopheline carrier and its habits** ... 10
3. **Endemicity—**
(a) Distribution and prevalence of malaria ... 11
(b) Malaria in relation to general morbidity ... 14
III. EPIDEMIC OF 1934-35 ... 18
1. **The epidemic area—**
(a) Boundaries ... 18
(b) Relationship to administrative areas ... 19
(c) Relationship to wet zone and river basins ... 19
(d) Relationship to population ... 20
(e) Relationship to endemic malaria ... 20
2. **Conditions preceding and during the epidemic—**
(a) Meteorological conditions and state of rivers ... 20
(b) Anopheline prevalence and infectivity ... 22
(c) Economic factors ... 26
3. **The start and course of the epidemic—**
(a) The epidemic by river basins ... 29
(b) The epidemic by districts ... 31
(c) Malaria in Colombo and other coastal towns ... 36
(d) Morbidity outside the epidemic districts ... 37
4. **Mortality during the epidemic—**
(a) Effect on general death rate ... 39
(b) Mortality by districts ... 39
(c) Age distribution of deaths... 43
IV. CLINICAL ... 46
1. **The malaria parasite** ... 46
2. **Clinical description of cases** ... 48
3. **Treatment** ... 53
4. **Pathology** ... 55
V. MEDICAL ORGANIZATION ... 56
1. **Normal organization** ... 56
2. **Emergency organization—** ... 57
(a) Official ... 57
(b) Voluntary ... 60
(c) General relief ... 62
3. **Anti-mosquito measures** ... 62
VI. PREVIOUS EPIDEMICS OF MALARIA ... 64
1. **Epidemics before 1900** ... 65
2. **Epidemics since 1900** ... 66
VII. SUMMARY ... 68
REFERENCES ... 70
BIBLIOGRAPHY ... 70
APPENDICES ... 71
1. List of contributors ... 71
2. Memorandum on areas likely to suffer from malaria during and after rainfall in late 1934 ... 71
3. Action to be taken in anticipation of recrudescence ... 72
4. Note on atebrin mussonate ... 73
5. Instructions to officers ... 79
6. Duties of Sanitary Inspectors in areas where malaria is epidemic ... 79
7. Instructions *re* issue of malted milk ... 79
8. Standard tonic mixture ... 80
9. Latrines for temporary hospitals and convalescent homes ... 80
10. Issue of quinine to estates ... 80
11. Conditions for temporary hospitals and convalescent homes ... 81
12. Medical work in schools reopened in bad malarious areas ... 81
13. (a) Instructions to Medical Officers of Health regarding the Malaria (Epidemic) Regulations ... 81
(b) Duties of temporary Assistant Sanitary Inspectors ... 82
14. Draft of Anti-mosquito Ordinance ... 82
15. Malaria (Epidemic) Regulations ... 86
16. Report on 1906 epidemic ... 87
17. Report on the epidemic to the Eastern Bureau ... 87
18. Press communiques ... 88
INDEX. ... 95

LIST OF TABLES.

No. PAGE
1. Monthly mean temperatures during 1934 with offsets from average ... 8
2. Monthly mean relative humidity during 1934, dry- and wet-bulb readings, with offsets from average ... 9
3. Monthly mean relative humidity during 1934 from minimum dry- and wet-bulb readings, with offsets from average ... 9
4. Mean monthly rainfall, 1911-1930, at stations in wet and dry zones ... 10
5. Endemic prevalence of malaria in Ceylon, spleen rates and parasite rates by Provinces, July, 1921, to July, 1922 ... 12
6. Spleen rates in principal towns of Ceylon during 1922-24 ... 13
7. (a) Normal seasonal morbidity in Ceylon, chiefly dry zone ... 15
(b) Normal seasonal morbidity, chiefly wet zone ... 16
(c) Normal seasonal morbidity, irregular peaks ... 17
8. Estimated population of epidemic area ... 20
9. Rainfall during the drought, April to September, 1934 ... 21
10. Natural infections in *A. culicifacies*. Epidemic zone, November, 1934, to May, 1935 ... 23
11. Relative prevalence of Anopheline larvae, December, 1934, to February, 1935 ... 24
12. Relative prevalence of *A. culicifacies* (larvae) in relation to epidemic conditions, December, 1934, to February, 1935 ... 24
13. Prevalence of *A. culicifacies* in river basins, December, 1934, to February, 1935 ... 25
14. Date of first appearance of epidemic at Government hospitals and dispensaries in each district ... 28
15. Weekly attendances at Government hospitals and dispensaries in five river basins in epidemic area, August, 1934, to April, 1935 ... 29
16. Weekly attendances at Government hospitals and dispensaries in each district in epidemic area, August, 1934, to April, 1935 ... 31
17. (a) Weekly attendances in Kurunegala District, August, 1934, to April, 1935, showing rise and development of the epidemic ... 33
(b) Weekly attendances in Kegalla District, August, 1934, to April, 1935, showing rise and development of the epidemic ... 34
18. Total attendances at Government hospitals and dispensaries during November, 1934, to April, 1935, compared with "expected" attendances ... 35
19. (a) Monthly totals of actual and "expected" attendances at Government hospitals and dispensaries in each district in the epidemic area, July, 1934, to April, 1935 ... [illegible]
(b) In each district outside the epidemic area ... 38
20. Actual and "expected" deaths by months in districts affected by the epidemic, November, 1934, to April, 1935 ... 40
21. (a) Monthly mortality rates, November, 1934, to April, 1935, in districts in the epidemic area ... 40
(b) Monthly mortality rates, November, 1934, to April, 1935, in districts outside the epidemic area 41
22. Deaths by months and mortality rates, November, 1934, to April, 1935, in headman's division of each district in the epidemic area ... 41
23. Infant mortality rates in each district during last quarter of 1934 and first quarter of 1935 combined 44
24. (a) Death rates at ages for the combined quarters, 4th quarter, 1934, and 1st quarter, 1935, in districts in epidemic area ... 45
(b) Death rates at ages for the combined quarters, 4th quarter, 1934, and 1st quarter, 1935, in districts outside epidemic area ... 46
25. Summary of the returns of blood films positive for malaria, November, 1934, to March, 1935 ... 47
26. Hospitals in the epidemic area, bed strength and in-patients ... 59
27. Government temporary hospitals, date of opening and accommodation ... 60
28. Equipment for a temporary hospital ... 60
29. Comparison of the mortality in Provinces and Districts in Ceylon during the epidemic years of 1906 and 1911 ... 67

LIST OF PHOTOGRAPHS.

1. Sand-pools in bed of Maha-oya, forming suitable breeding places for *A. culicifacies* (December, 1934).
2. Rock-pools in Maha-oya, forming suitable breeding places for *A. culicifacies* (December, 1934).
3. Mahaweli-ganga at Katugastota, 23rd February, 1935.
4. Mahaweli-ganga at Haragama (on road to Hanguranketa) January, 1935.
5. Mahaweli-ganga, ½ mile below Haragama, January, 1935.
6. Tributary of Mahaweli-ganga flowing after local rain, February, 1934.
7. Oedema in a child.
8. Patients at a temporary treatment centre (December, 1934).
9. Patients at Narammala Dispensary.
10. Temporary kitchen for out-patients.
11. Transport of patients by bullock cart.
12. Improvised stretcher (Kandy District, December, 1934).
13. Anti-anopheline work on the Maha-oya river—18th January, 1935.
14 Oiling work in Kospotu-oya, Kurunegala—20th February, 1935.
15. Canalising and oiling of Kospotu-oya—20th February, 1935.

The report tabled in the House of Commons was the *Report on the malaria epidemic in Ceylon in 1934-35: together with a scheme for the control of malaria in the island* by Colonel CA Gill (Ceylon Government Press, Colombo, 1935). The total numbers of deaths from malaria in Ceylon were 1,681 in 1932, 1,409 in 1933, and during the period from September 1934 to December 1935, were 100,000 in excess of the average of the five preceding similar periods. Details of the epidemic can be found in articles in the *British Medical Journal* (1935:1:209, 590, 1001-1002, and 1935:2:552, 1001-1002, 1015-1017).

Charts and graphs

Hilda Woods did learn, and “construction of charts and graphs” was the title of the third chapter of her book with William Russell *An Introduction to Medical Statistics* (1931). She also published a paper on the graphic method of graduation in life-tables (Woods HM. A note on the graphic method of graduation in the construction of life tables. *Lancet* 1929;I;941-942.)

Doddershall, Quainton

Quainton (old English *cwen + tun*, 'the queen's farmstead or estate') is a parish just over six miles north west of Aylesbury in Buckinghamshire, Doddershall is a hamlet within this parish.

Drummond, Professor Sir Jack Cecil, FRIC, FRS (1891-1952)

A distinguished biochemist who was appointed in October 1939 as chief adviser on food contamination in the Ministry of Food. In April 1940 Drummond produced a plan for the distribution of food based on "sound nutritional principles". He recognised that rationing was the perfect opportunity to attack "dietetic ignorance" and that, if successful, he would be able not just to maintain but to improve the nation's health. Thanks to Drummond's advice, the effect of rationing was to introduce more protein and vitamins to the diet of the poorest, while the better off were obliged to cut their consumption of meat, fats, sugar and eggs. Follow-up studies after the war showed that, despite rationing and the stresses of war, the population's health had improved. He was murdered, together with his wife and 10-year old daughter, on the night of 4th August 1952, near Lurs, a village in the Basses-Alpes region of Southern France.

Dunhill, Sir Thomas Peel, CVO, CMG, KCVO, GCVO (1876-1957)
A leading Australian surgeon who in 1907 undertook ground-breaking treatment for exopthalmic goitre by removal of the thyroid gland. Within three years he had carried out over 312 similar operations with a mortality rate of only 1%. This was an outstanding achievement as the mortality rate of thyroidectomies in London was at least 30%. He was one of the most honoured surgeons of the time.

Ellerker College, Richmond, Surrey
Founded in 1881 at 48 Richmond Hill in South London it was built originally as the family home of Sir John and Lady Houblon, descendents of a Huguenot family who fled to this country about 1567 during the Alva persecutions; he was the first Governor of the Bank of England in 1694 and Lord Mayor of London in 1696. The College changed its name in 1931 to become The Old Vicarage School and exists today as an independent preparatory school for girls aged 4 to 11 years; initially it was a boarding school for older girls.

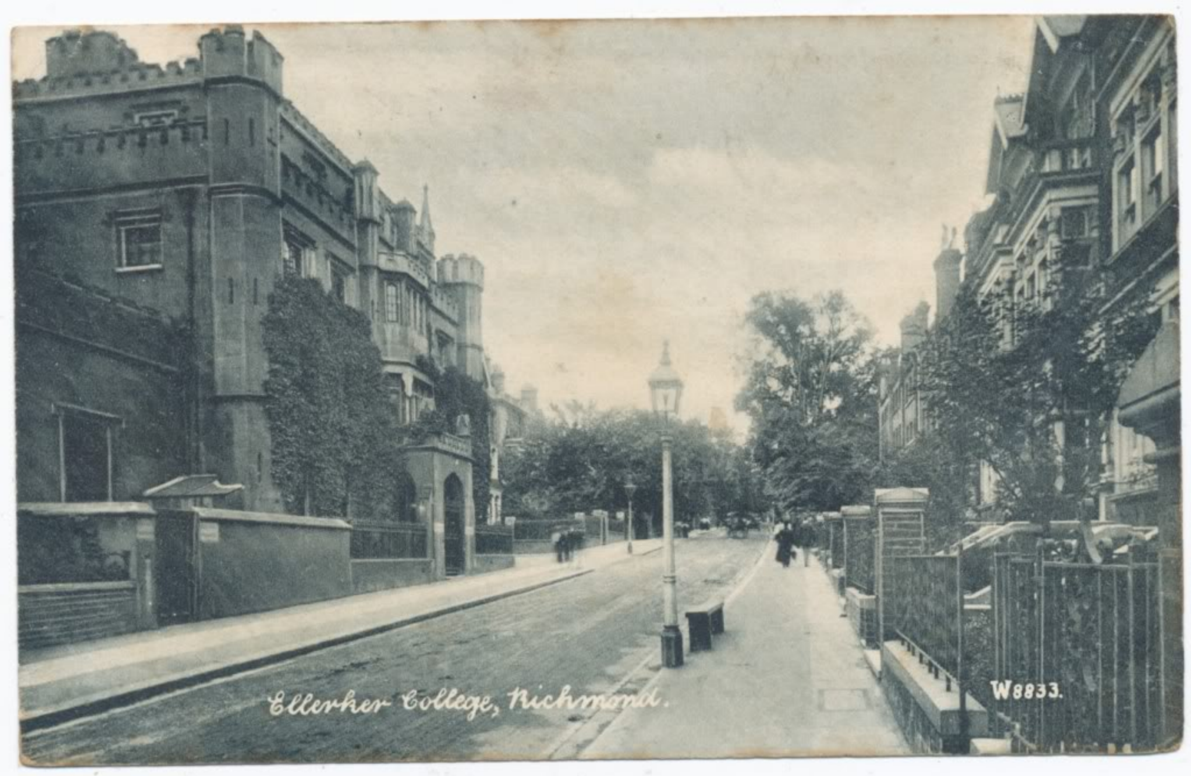

The postcard picture above was taken in 1908 when Hilda Woods was attending the College which is the building on the left; its Gothic architecture and turrets are plainly visible.

Factories, statistics from
This work culminated in Greenwood's publication in the Medical Research Committee's Special Report Series, no. 16, *A report on the causes of wastage of labour in munitions factories employing women* (His Majesty's Stationery Office, London, 1918) in which he acknowledges the help of his staff, "Finally, it is my pleasant duty to acknowledge the valuable assistance rendered by my co-workers, Miss Thompson, Miss Wilcox and Miss Woods, upon whom has fallen nearly all the labour of collecting and much of the labour of analysing the data."

First dietary survey
Hilda Fowke's remark was "Some of us spent a good many hours trying to ensure that the first dietary survey made by the Ministry of Food should fulfil the required conditions but, when the time came, the most careful planning was upset by 3 factors, the blitz, evacuation, and the absence at work of the housewife." This indicates that she was a member of the team that carried out this first National Food Survey (NFS) in 1940 which was set up to provide information on household food purchases and the nutritional value of the domestic diet for the 'urban working class'. In 1950 the coverage of the survey was widened to the population of Great Britain. It closed in 2000 by when it had bcome the longest-running continuous survey of household food consumption and expenditure in the world.

Flapper
In the 1920s a term applied to young Western women who flaunted their disdain for what was then considered acceptable behaviour. They wore short skirts, bobbed their hair and listened to jazz. and were seen as brash for wearing excessive makeup, drinking, smoking, driving cars and otherwise flouting social norms. Flappers had their origins in the period of liberalism, social and political turbulence and increased transatlantic cultural exchange that followed the end of the First World War. The term may also apply to the vigorous dancing (flapping) partly inspired by American jazz.

Hilda's description of herself as a "flapper" was as one who believed in equality between the sexes, and dancing the foxtrot. Greenwood uses the word in a descriptive, and most definitely not in a derogatory sense.

Fortified chocolate
Unfortunately there are few details of this randomised study beyond those reported in the BMJ paper. Searches by one of us (TJ) among the files for the Ministry of Food (MAF series) and the Ministry of Health (MH series) for the period 1938 to 1946 at the National Archives (Kew, Richmond, Surrey) have found only isolated copies of her paper (in files MH 56/232 and MH 56/236), a reference to the paper in a summary table of dietary surveys other than the National Food Survey (file MAF 256/164), and a reference to the study in a memorandum by Drummond (in file MAF 152/146) which states that the girls were residing in orphanages where Poor Law children are received. There is no sign of the "report in full" mentioned in the footnote to the published paper.

Hilda Fowke's randomised study was carried out between February 1941 and April 1942, roughly the same time (November 1941 to August 1942) as the vitamin feeding tests carried out by the Ministry of Health in 1,242 school children in Ipswich, Glossop, and London. These children were divided at random into two equal groups according to school, age, and sex with one group receiving a vitamin capsule every school day, and the other a capsule containing a similar quantity of archis oil. (BMJ 15 Jan 1944:77-78). Ministry of Health documents (MH 56/232) reveal that children with odd numbers received the experimental capsules while those with even numbers received the control. There are no details of how the numbers were assigned to the children. We conjecture that these studies were performed in a similar way and that the children were not individually randomised.

Fowke, Roger Warburton (1880-1934)
Roger Fowke was born in Kandy, Ceylon, the fourth son of Philip Francis Fowke (1846 – 1897) and Susan French Gray (1850 – 1916).

Gear, James Henderson Sutherland (1905-1994)
One of South Africa's greatest doctors and a pioneer in the prevention and control of infectious disease. He became Director of the South Africa Institute for Medical Research and also Director of the Poliomyelitis Research Institute in Johannesburg.

Girls High School, Northampton
A private selective non-boarding school for girls in Derngate, Northampton founded in 1878 by a committee of local church people.

Hardwick(e)
A village about four miles north of Aylesbury in Buckinghamshire, and Hilda Fowke's address in 1945, when she attended the 18th meeting of the Nutrition Society, is given as the Manor House. Its population in 1951 was 167. Hardwick is a common name in England, of Anglo Saxon origin, meaning 'livestock farm'.

Home Office, London
The Home Office was created in 1792 and is responsible for the domestic civil affairs of the UK; it is headed by the Home Secretary, a member of the UK government. Its initial responsibilities were answering petitions and addresses sent to the King and advising him on Royal grants, warrants and commissions, the exercise of Royal Prerogative, issuing instructions on behalf of the King to officers of the Crown, Lords Lieutenant and magistrates, mainly concerning law and order, operation of the secret service within the UK, protecting the public, and safeguarding the rights and liberties of individuals; these responsibilities were changed over the years that followed. In 1916 the Home Secretary was Sir Edward Troup.

.

India Office, London
The India Office (1858–1947) was the department of the British government responsible for the direct administration of the territories under the British Empire which included areas today known as sovereign and independent nations of Bangladesh, Burma, India, and Pakistan, including major parts of the areas at different times, covering Southeast Asia, Central Asia, the Middle East, and parts of Africa. The India Office covered all activities concerning their administration before 1947. It was created as a result of the widespread commercial activities of the East India

Company and headed by the Secretary of State for India, who was a member of the British Cabinet.

Indian ink
A black ink once widely used for writing, printing, and drawing. It is composed of a variety of fine soot known as lampblack, combined with water to form a liquid. A binding agent such as gelatin or shellac may also be added, to make the ink more durable once dried. It was called masi in India where it has been in use since at least the 4th century BC

Junior Oxford and Cambridge School Certificate
In 1873 the universities of Oxford and Cambridge formed the Joint Board of Oxford and Cambridge Schools Examinations. Their Junior School Certificate examination was taken at 16 years of age.

Keppel Street, London
The London School of Hygiene and Tropical Medicine is located here.

Lister Institute of Preventive Medicine
The Lister Institute (named after Dr Joseph Lister) was founded at Chelsea in 1891 as a voluntary non-profit making body; it was originally called the British Institute of Preventive Medicine, and then the Jenner Institute. The intentions of the founders were the preparation of vaccines and sera, stemming from the work of Koch and Pasteur, with particular reference to rabies, and secondly, to train medical officers of health. Early difficulties were due to inadequate finances and the fervid antagonism of the antivivisectionists. The Institute conducted a wide variety of research in bacteriology, biochemistry, nutrition, blood structure, vitamins, and Caisson disease, among others. It is currently a funding body.

Long, Frank Stevenson
Frank Stevenson Long, a lieutenant in the 11th battalion of the Essex Regiment, was killed in action on September 26th, 1915 in the Battle of Loos, one of the major British offensives mounted on the Western Front in 1915 from September 25th to October 13th. General Douglas Haig, then commander of the British First Army, directed the battle though his plans were limited by a shortage of artillery ammunition. The battle marked the first time the British used poison gas during the war. British losses amounted to 60,000, total allied losses to 250,000, and German losses to 50,000. Long published a paper *The velocity of addition of alkyl bromides to cyclic tertiary bases* in Journal of the Chemical Society Transactions (1911:99:2164-2171).

LRAM
Licentiate of the Royal Academy of Music is a professional diploma formerly open to both internal students of the Royal Academy of Music and external candidates in voice, keyboard and orchestral instruments and guitar, as well as conducting and other musical disciplines. Those awarded the diploma are entitled to use the post-nominal letters LRAM and to wear the appropriate academic dress: black bachelor’s gown with a simply shaped scarlet silk hood.

Lyons’ Corner Houses

These were chains of tea shops opened by J Lyons & Co in 1894, and of Lyons' Corner Houses from 1909 in the West End of London. The tea shops were slightly more up-market than those run by the Aerated Bread Company (ABC). They were notable for their interior design and working class aspect. The tea shops always had a bakery counter at the front, and a table service by uniformed waitresses, known as 'Nippies'; they converted to cafeteria service after 1945. Corner Houses remained until 1977.

Maclennan, Norman Macpherson, MD, MB. Ch.B, DPH, DTM&H (1895-?)
A distinguished bacteriologist who in 1923 was appointed lecturer in bacteriology at the St. Andrew's Institute for Clinical Research. He later worked in Kenya before appointment as Senior Medical Officer, Department of Health in Palestine.

Medical Research Council (MRC) and Department of Health amalgamation
The Medical Research Council (MRC) was established by Royal Charter in 1920, (its fore-runner the Medical Research Committee in 1913) funded by, but otherwise independent of, Government. By contrast the Department of Health (DoH) is part of the Ministry of Health, one of the largest subdivisions of government that includes the National Health Service. Amalgamation of the two has been debated regularly since their creation.

Ministry of Munitions, Welfare section
The Ministry of Munitions was a British government department created during the First World War to oversee and co-ordinate the production and distribution of munitions for the war effort. The position was created in response to the shell crisis of 1915 when there was much public criticism of the shortage of shells available. The first Minister of Munitions up to 9 July 1916 was David Lloyd George who later became Prime Minister. The First World War demanded rapid production of munitions, so the Ministry devised training schemes aimed at producing competent machine operators in under three months. In 1917 the emphasis shifted to skill training for (mainly) disabled ex-servicemen, to enable them to gain both employment and trade union membership

The Welfare Section where Greenwood worked was directed by Benjamin Seebohm Rowntree (1871-1954) the third child of Quaker chocolate manufacturer Joseph Rowntree and Emma Seebohm. He was educated at the York Quaker Boarding School and Owens College, Manchester, where he studied for five terms focusing on chemistry. When he returned to York he started working in his father's chocolate factory where he used his knowledge of chemistry to carry out research and laboratory testing. On Sundays he started teaching at the York Adult School which he continued for twenty years. His interaction with members of the working class at the Adult school combined with a visit to Newcastle in 1895, in which he was shown the living conditions of the poor first hand, made him determined to look into the problem of poverty. In 1897 he was appointed as a director of his father's successful business which allowed him time to embark on his first investigation of poverty in York. During the First World War he was the director of the welfare department of the Ministry of Munitions and in 1917 became a member of the reconstruction committee which later became the Ministry of Reconstruction. He became chairman of Rowntrees in 1923 a post he held until 1941.

Monty
Presumably a reference to *The Count of Monte Cristo* by Alexandre Dumas published in 1844.

MRC
See *Medical Research Council and Department of Health amalgamation*

National Institute for Medical Research (NIMR), Hampstead
The Medical Research Committee, founded in 1913, was immediately charged with establishing a central research institute in London. Later that year premises at Mount Vernon Hospital, Hampstead were acquired and the National Institute for Medical Research was founded. However, the outbreak of World War I postponed occupation of the building, although senior staff were appointed and began work. By 1920 the Institute was fully operational and remained so for 30 years until the move to its current location at Mill Hill, in North London. The original Institute, under the directorship of Sir Henry Dale, had three divisions: bacteriology, biochemistry and pharmacology, and applied physiology.

Newbold, Ethel May (1882-1933)
Although ten years older than Hilda Woods, her interest in statistics also started during the First World War while working for the Ministry of Munitions. In 1926 she studied at the Galton Laboratory, University College, London, was a member of the MRC Statistics Committee and collaborated frequently with Greenwood. She was the first woman to receive the Guy medal of the Royal Statistical Society (1928), but suffered a breakdown in that year and resigned from LSHTM on account of ill health in 1929.

Portia
A character in Shakespeare's *The Merchant of Venice* described as the first of his women who unite in beautiful proportion, intellectual power, high and refined, with unrestrained ardour of the heart. Portia's role as an advocate is remembered in the naming of the Portia Law School in Boston, USA. We assume Greenwood's form of address is an acknowledgement of Hilda Woods' role as an advocate.

Retro-sternal goitre
A thyroid gland enlarged downwards into the chest, sometimes due to iodine deficiency. Compressive symptoms may cause diagnostic problems and the selection of a surgical approach is sometimes difficult.

Royal Academy of Music, Marylebone Road, London
Britain's oldest degree-granting music school and a constituent college of the University of London since 1999. The Academy was founded by Lord Burghersh in 1822 with the help and ideas of the French harpist and composer Nicolas Bochsa and in 1830 was granted a Royal Charter by King George IV. The Principal when Hilda Woods attended was the Scottish composer Sir Alexander Campbell Mackenzie (1847-1935).

Senior Cambridge Certificate
The Cambridge Local Examinations were introduced in 1858 by the University of Cambridge Local Examinations Syndicate (UCLES) and consisted of the Junior Local

Examination for pupils under sixteen and the Senior Local Examination for those under eighteen. These eventually became the School Certificate and the Higher School Certificate.

Shilling
Unit of currency in UK until decimalisation in 1971; there were twelve pence in a shilling, and twenty shillings in one pound sterling. Several amounts are mentioned in the text and, based on the retail price index, 15/- (fifteen shillings and no pence) in 1916 is equivalent to about £38 today; 7/6 to about £19; 25/- to about £63; and 27/6 to £70. Sixpence in 1916 is now worth £1.26, and £150 is now worth £7.600.

Sickness Returns
These were weekly records of sickness introduced around 1870 but only in sporadic locations to provide more useful details of morbidity than could be obtained from the mortality records that had been collected since 1837. In 1877 the first local Act for enforcing compulsory notification of the chief infectious diseases came into operation in Bolton, Lancashire, and following the Infectious Diseases (Notification) Act in 1889, was followed by rapid adoption by urban and rural authorities throughout England. However, by 1896 the Act applied to only five-sixths of the population. (See Newsholme *A national system of notification and registration of sickness*, (Journal of the Royal Statistical Society 1896:59:1-37)). The statutory requirement for the notification of certain infectious diseases came into being towards the end of the 19th Century.

Diseases such as cholera, diphtheria, smallpox and typhoid had to be reported in London from 1891 and in the rest of England and Wales from 1899. The list of diseases has been increased over the decades and now stands at about 30. The prime purpose of the notifications system is speed in detecting possible outbreaks and epidemics. Accuracy of diagnosis is secondary, and since 1968 clinical suspicion of a notifiable infection is all that is required.

Suffolk
“when we lived in Suffolk”. This is the beautiful, historic village of Clare situated in the rolling Suffolk countryside. It is an old wool town with a fascinating history, many interesting places to visit, and a wide range of shops, antique outlets, cafes and pubs.

Tuberculosis incidence and industrial conditions
This work appears to have culminated in Greenwood’s report with Tebb, *An inquiry into the prevalence and aetiology of tuberculosis among industrial workers, with special reference to female munition workers* (Medical Research Council Special Report Series, no. 22, His Majesty’s Stationery Office, London, 1919). Greenwood published articles on tuberculosis throughout his career.

Wastage of Labour
See *Factories, Statistics from*

Whitchurch and District Nursing Association

This is Whitchurch in Buckinghamshire, about six miles north of Aylesbury and four miles northeast of Quainton. The Whitchurch Oving and Pitchcott Nursing Association was founded in 1908 and renamed the Whitchurch and District Nursing Association in 1938 following the inclusion of Hardwick and Weedon. Hilda Fowke was treasurer from 1939 to April 1940 when she resigned to take up work with government (presumably the nutrition studies for the Ministry of Food) but was joint president from 1945 to 1946. It is now called the Whitchurch and Oving District Nursing Association.

Worcestershire Artillery Regiment

This is likely to be the 1st (Worcester) Battery of the 241 Brigade of the Royal Field Artillery (RFA) which lists WA Woods among its members, and should not be confused with the Worcestershire Regiment. Prior to October 1916 it was known as the 2nd South Midland Brigade, RFA.

Yule's *Elements of Statistics*

We are unaware of a text with this title by Yule. The reference must be either to Bowley's text with this title first published in 1901, or to Yule's *An Introduction to the Theory of Statistics* first published in 1911.
